# Hybrid Modality Fusion of Planar Scintigrams and CT Topograms to Localize Sentinel Lymph Nodes in Breast Lymphoscintigraphy: Technical Description and Phantom Studies

**DOI:** 10.1155/2011/298102

**Published:** 2010-12-14

**Authors:** Renée L. Dickinson, William D. Erwin, Donna M. Stevens, Luc M. Bidaut, Martha V. Mar, Homer A. Macapinlac, Richard E. Wendt

**Affiliations:** ^1^Graduate School of Biomedical Sciences, The University of Texas Health Sciences Center at Houston, Houston, TX 77030, USA; ^2^Division of Diagnostic Imaging, Department of Imaging Physics, The University of Texas M. D. Anderson Cancer Center, Houston, TX 77030, USA; ^3^Department of Radiology, University of Washington, Seattle, Washington 98195, USA; ^4^Department of Radiology, Oregon Health and Science University, Portland, OR 97239, USA; ^5^Centre for Oncology and Molecular Medicine (COMM), University of Dundee—Ninewells Hospital and Medical School, Dundee DD1 9SY, Scotland; ^6^Division of Diagnostic Imaging, Department of Nuclear Medicine, The University of Texas M. D. Anderson Cancer Center, Houston, TX 77030, USA

## Abstract

Lymphoscintigraphy is a nuclear medicine procedure that is used to detect sentinel lymph nodes (SLNs). This project sought to investigate fusion of planar scintigrams with CT topograms as a means of improving the anatomic reference for the SLN localization. Heretofore, the most common lymphoscintigraphy localization method has been backlighting with a ^57^Co sheet source. Currently, the most precise method of localization through hybrid SPECT/CT increases the patient absorbed dose by a factor of 34 to 585 (depending on the specific CT technique factors) over the conventional ^57^Co backlighting. The new approach described herein also uses a SPECT/CT scanner, which provides mechanically aligned planar scintigram and CT topogram data sets, but only increases the dose by a factor of two over that from ^57^Co backlighting. Planar nuclear medicine image fusion with CT topograms has been proven feasible and offers a clinically suitable compromise between improved anatomic details and minimally increased radiation dose.

## 1. Introduction

An essential step in the procedure for sentinel lymph node (SLN) biopsy is to locate the first-echelon node of the drainage basin. Lymphoscintigraphy is a minimally invasive diagnostic tool for mapping the SLN. After injecting the patient with sulfur colloid radiolabeled with ^99m^Tc in locations proximal to a tumor site and waiting for lymphatic drainage to occur, the lymphatic drainage pathway from the primary injection site may be imaged with a gamma camera [1]. 

Interpretation of lymphoscintigrams is hindered by the absence of anatomical landmarks in the scintigraphy image. To partly cope with this limitation, it is customary to acquire additional transmission images via a backlighting ^57^Co sheet source in order to facilitate the anatomic localization of the nodes seen on the scintigraphy image. While the backlighting imaging technique creates an outline of the patient's body, it depicts no internal landmarks.

The introduction of hybrid single photon emission computed tomography with computed tomography (SPECT/CT) systems into clinical practice presents an opportunity to improve the spatial localization in lymphoscintigraphy [2]. Improvement in the localization technique could potentially help the surgeon prepare for sentinel node biopsy (SNB) operations, for example, when SLNs lie in the internal mammary chain where bony anatomy necessitates pinpointing the location of a node before incision. Single photon emission computed tomography and computed tomography image fusion (known as SPECT/CT) provides accurate three-dimensional (3D) localization of lymph nodes at the cost of a significant increase in the absorbed dose to the patient for the overall lymphoscintigraphy and CT procedure. At M.D. Anderson Cancer Center (MDACC), SPECT/CT is routinely performed for lymphoscintigraphy of the head and neck because of the intricate anatomy. For those situations in which planar scintigrams would suffice, we have developed a technique for fusing the CT topograms (also known as the CT “scout” or pilot image) with planar lymphoscintigrams in order to improve the localization of SLNs with better anatomical information than would be offered by ^57^Co backlighting, but with less absorbed dose to the patient than would be imparted by CT localization. 

**Figure 1:**
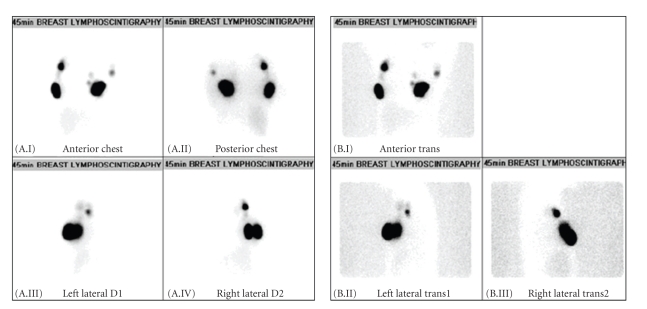
Traditional breast lymphoscintigraphy technique with ^57^Co-backlighting for localization. The four images on the left (A.I–A.IV) are emission images for a bi-lateral lymphoscintigraphic study. The current protocol for localizing sentinel nodes is to backlight the patient using a ^57^Co sheet source to create a silhouette of the patient. The anterior (B.I), left lateral (B.II), and right lateral emission + ^57^Co transmission images (B.III) are shown on the right.

Conventional Method for Node Localization—^57^Co Backlighting: the most common and traditional localization method for breast lymphoscintigraphy is the use of a low-activity ^57^Co sheet source to backlight the patient (Figure 1) [1, 3, 4]. Following the acquisition of a ten-minute static anterior emission image, a ^57^Co sheet source is placed below the patient on the frame of the posterior camera of a dual-head gamma camera. A three-minute static anterior patient silhouette (or backlit) image is acquired that allows the physician to locate the node in relation to the outline of the patient. Such an approach aids in assessing whether lymphatic vessels from the tumor site in the breast are draining to the internal mammary nodal basin or to the axillary nodal basin. 

A lateral emission image is used to estimate the anterior depth of the node. The technical details of acquiring the lateral images vary among institutions. One approach is to use a chair positioned adjacent to the bed to prop the sheet source vertically next to the patient [1, 5–7]. In another case, the source is placed on top of the detector of the gamma camera below the patient for both the anterior and lateral images because the lateral images are acquired by rotating the patient to a lateral decubitus position while leaving the gamma camera gantry in the “zero-degree” orientation. 

 The absorbed dose to the patient is kept low by placing a ^57^Co sheet source of relatively low activity, 74–185 MBq (2–5 mCi), on the distal side of the patient for a maximum of two to three minutes, which provides sufficient photon fluence to obtain an adequate outline of the patient. Clarke, Notghi, and Harding reported that ^57^Co backlighting adds approximately 25% to the absorbed dose to the patient from a ^99m^Tc-nanocolloid injection [8]. 

SPECT/CT for Lymphoscintigraphy: the great promise of hybrid SPECT/CT [9] and PET/CT [10] cameras is the mechanical registration of emission data with a highly detailed X-ray computed tomogram, thereby combining the sensitivity and specificity of a radiopharmaceutical study with the high resolution morphological information of a CT scan. The value of SPECT/CT for SLN identification and localization has been described in several reports [11–14]. In special instances, SPECT imaging allows for improved detectability and interpretation of lymphatic drainage. Contamination, nodes close to the injection site, and overweight patients are three noted instances in which SLN identification and localization are better with SPECT than with standard planar methods [15, 16]. Overall, the detectability studies comparing planar imaging and SPECT for the general population do not indicate a compelling reason to migrate to SPECT for breast lymphoscintigraphy studies. This is in contrast to protocols such as head and neck lymphoscintigraphy that nearly always greatly benefit from using SPECT/CT for SLNs identification and localization, for example, because the anatomy in that area is much more complex than that of the breast [17].

## 2. Materials and Methods

The development of our topogram fusion technique began with a retrospective chart review that was used as a guide toward the design and implementation of a phantom that would realistically mimic a patient undergoing a breast lymphoscintigraphic exam. Topograms of a simple alignment phantom were analyzed to assess the spatial distortion effects of the fan-beam projection geometry. A protocol to acquire and process topograms and scintigrams in anteroposterior and lateral views was implemented. A computer program was written to process and prepare both topogram and scintigram prior to their fusion. Phantom data were acquired, prepared, and fused for the characterization and validation of our approach. In addition, data were acquired and doses calculated to estimate the absorbed doses to patients from the ^57^Co-backlighting, topogram fusion, and SPECT/CT approaches, respectively. 

### 2.1. Retrospective Chart Review

A retrospective review of lymphoscintigrams of 200 patients was approved by the Institutional Review Board (IRB) at U.T. M. D. Anderson Cancer Center, where the study was conducted. Although physiological processes vary from individual to individual (affecting the drainage rate and amount of Tc-99m sulfur colloid collected in an SLN), the apparent activity of the node (*A*_*N*_) in a subset of the 200 patients reviewed was calculated using the total counts in the injection site (*N*_IS_), the total counts in the SLN (*N*_*n*_), and the known amount of injected activity (*A*_IS_).

(1) $$A_{N}=A_{\text{IS}}\cdot \frac{N_{n}}{N_{\text{IS}}}.$$

Calculations made from regions of interest around SLNs in the clinical images estimated that the radioactivity in a typical lymph node was 1.1–1.8 MBq (30–50 *μ*Ci) of Tc-99m. This level of activity is consistent with estimates in the literature [15–17].

### 2.2. Quantifying Spatial Distortion

**Figure 2:**
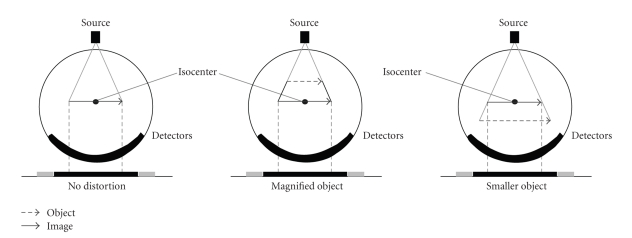
Diagram of object magnification and minification for objects in different coronal planes relative to isocenter. Topograms differ from projection radiographs because the topogram is calibrated such that a “virtual” detector lies at a plane through the isocenter. The gray arrow represents a real object that is projected to isocenter during topogram reconstruction. This reconstruction technique causes objects that are physically closer to the X-ray source, relative to the gantry isocenter, to appear larger in the image, and vice versa when the object is physically closer than isocenter to the detectors (unlike projection radiographs, where objects closest to the detector are least affected by distortion).

Fusion of a lymphoscintigram that is, a projection along parallel ray paths—with a CT to pogram that is, a projection with fan-beam geometry—is complicated by the inherent misregistration between the two projection geometries. Topogram geometry differs from the geometry of projection radiographs because a “virtual” detector lies at a plane through isocenter for the acquisition of topograms. As in projection radiography, the fan-beam geometry causes objects closer to the x-ray source than the gantry isocenter to appear magnified laterally in the detected image. Unlike radiographs, where objects closest to the detector appear less distorted in the image than objects closer to the x-ray source, objects in topogram acquisitions that are closer to the x-ray detectors than the gantry isocenter will appear smaller in the image due to reconstruction software for topograms. Figure 2 summarizes the various situations leading to spatial distortion.

The spatial distortion due to the geometry of the fan-beam affects the location of objects in the topogram in both the anteroposterior and lateral directions. There is no distortion along the long axis of the patient because the CT topogram beam is tightly collimated so the edges are nearly parallel in the cranio-caudal direction. As a special case, small objects at the isocenter of the gantry remain at the center of the imaging plane and suffer minimal magnification and geometric distortion in contrast to objects located away from the gantry isocenter. 

The amount of magnification in the lateral direction varies as a function of the distance of the coronal plane (of a supine patient) to the gantry isocenter axis. For vertical planes perpendicular to the patient couch (sagittal planes of a supine patient), the lateral distortion is the difference between the actual lateral distance of an object relative to the gantry isocenter and the measured lateral distance of that object to the central axis of the image. The degree of magnification and geometric distortion of that object varies as a function of the distance of the sagittal plane to the gantry isocenter axis.

To compute the magnitude of the distortion of objects at different coronal and lateral distances (assuming a supine patient orientation), a point source phantom was constructed. Six 1.5 mm diameter skin markers used for mammography, consisting of a metal bead with adhesive tab, were positioned along one surface at 2.5 cm, 5.0 cm, 10 cm, 15 cm, and 20 cm from the center of a fillable sheet source phantom. The central axis of the rigid phantom was aligned with the gantry lasers of the SPECT/CT scanner and the height of the table was initially adjusted so that the center of the beads was at the isocenter. Topograms of the phantom were acquired at different table heights between 6.7 cm and 14.0 cm, including the table height position corresponding to the beads at isocenter (table position = 0 cm). The spatial distortion of point objects at different anteroposterior and lateral positions was quantified by using the digital ruler tool in the gamma camera workstation software (e.soft, Siemens Medical Solutions USA, Hoffman Estates, IL) to measure the lateral distance of the bead from the gantry isocenter axis in the topogram image. The lateral distortion (or offset) for each point was computed as the difference between the measured distance and the known distance from the isocenter axis.

### 2.3. Topogram Localization Protocol for Breast Lymphoscintigraphy

#### 2.3.1. Acquisition Workflow

The camera is configured with the detectors in a 180 degree, opposed-view orientation and static images are acquired in SPECT mode at zero and 90 degree gantry positions. The resulting four images (anterior, posterior, left lateral, and right lateral) are saved as separate static DICOM images in the same series. The emission images are acquired in a 256 × 256 matrix for ten-minutes per view at each camera position. Posterior/anterior (PA) and ipsilateral topograms are acquired using a 110 kV_p_, 20 mA beam. As patients on a SPECT/CT scanner would not move for the lateral images, the position and shape of the breast tissue are more similar to the configuration that the breasts will have at the time of surgery than if the patient were to rotate to the decubitus position for the lateral views [1].

Following image acquisition, the original scintigrams are adjusted for single-point registration and fusion to the topogram of the SLN as described below. The lateral magnification factor is applied to the scintigram to match the geometry of the topogram so that the fan-beam geometry of the topogram will remain consistent regardless of the location of an SLN. The original topogram is output as a new image series with an NM modality tag (rather than a CT modality tag) in the DICOM header, since the image fusion software we used would not permit fusing planar NM data with CT datasets. Fusion images of each scintigram/topogram pair are then created for subsequent review.

#### 2.3.2. Compensation for Geometric Distortion

A computer program was written in the Interactive Data Language (IDL, ITT Visual Solutions, Boulder, CO) to automate the process of adjusting the lateral magnification of each scintigram. The program was integrated into the image processing application environment of the gamma camera workstation (e.soft, Siemens Medical Solutions USA, Hoffman Estates, IL). The program accepts as inputs four DICOM images: anterior and ipsilateral planar lymphoscintigrams, and posterior/anterior and ipsilateral CT topograms. Assuming that the central pixel of the image corresponds to the isocenter of the gantry, the distance from the central pixel to the SLN in both the anterior and lateral images can be used to laterally magnify or minify the scintigrams to match the geometries of the topograms and scintigrams for a particular node location. The operator manually marks a desired feature such as an SLN on each of the two orthogonal lymphoscintigrams. The location of the SLN relative to the isocenter in the lateral lymphoscintigram is then used to apply the appropriate lateral magnification factor to the anterior lymphoscintigram. This is because the degree of distortion of objects in the posterior/anterior topogram is dependent upon the coronal plane in which the object lies. The depth of the SLN in the lateral lymphoscintigram gives the distance of the coronal plane from the isocenter and the appropriate amount of distortion correction can then be applied. A similar process is used to correct the lateral lymphoscintigram. The location of the node in the anterior lymphoscintigram provides the offset of the sagittal plane containing the node relative to the gantry isocenter. 

After the magnification factor has been applied, the program generates two new image series: a series of laterally stretched lymphoscintigrams and a series of CT topograms with NM modality tags. In cases where several nodes are visible and widely scattered, the scintigram can be adjusted differently and a separate fused image created for each one of the nodes. We chose to modify the lymphoscintigram rather than the topogram so that the geometry of the morphological information will retain a consistent fan-beam presentation.

### 2.4. Verification of Geometric Compensation Accuracy

#### 2.4.1. Point Source Phantom

A rigid-plane phantom was placed on the patient bed of the hybrid SPECT/CT system with three ^57^Co button sources positioned at various lateral distances from the central axis of the scanner. Static emission images and CT topograms were acquired in both the anterior and lateral views at several bed height positions to take into account different SLN anterior-posterior positions. The images were processed using the geometric compensation program and analyzed to compute the residual misregistration of each point source between the scintigram and the topogram.

#### 2.4.2. Anthropomorphic Thorax Phantom

**Figure 3:**
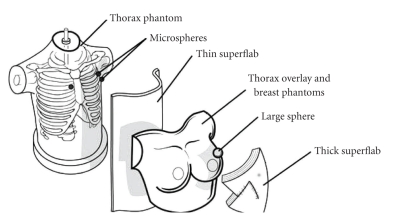
Phantom Setup for Breast Lymphoscintigraphy Studies. The micro spheres used to represent the lymph nodes are applied directly to the surface of the thorax phantom. 0.5 cm of thin superflab is placed over the nodes, then the chest overlay and breast phantoms are positioned. The 4.0 mL sphere used for the injection site is then embedded in a 2-3 cm thick piece of superflab that is placed on one of the breast phantoms.

In order to have a reproducible test object, a nuclear medicine anthropomorphic phantom (Radiology Support Devices, Inc, Long Beach, CA) was augmented in order to simulate a breast lymphoscintigraphy injection site and sentinel lymph nodes (Figure 3). Fillable spheres (Data Spectrum Corporation, Chapel Hill, NC), such as those used in the NEMA IEC Body Phantom, were used to mimic the node and injection sites. Lymph nodes typically are 4 to 6 mm in diameter. Therefore, fillable spheres of inner diameter 4.95 mm and volume 0.063 mL were used to represent SLNs in the phantom. Initial images of the phantom indicated that the previously estimated 1.1–1.8 MBq (30–50 *μ*Ci) nodal activity, determined by the chart review, was too high. A total activity in the node between 37 kBq and 370 kBq (1 and 10 *μ*Ci) was used, leading to a range of activity concentrations from 0.59–5.9 MBq per mL (16 to 160 *μ*Ci per mL). A fillable sphere of inner diameter 19.79 mm and volume 4.0 mL was used for the injection site. The sphere used for the injection site was filled with an absolute activity of 92.5 MBq (2.5 mCi); the volume concentration for the 4.0 mL sphere is thus 23.1 MBq per mL (0.625 mCi per mL).

Generally, the distance between the anterior surface of the breast and the injection site ranges from a few millimeters for a subcutaneous injection to about 3 cm for a perilesional injection. The distance between the injection site and the visualized node in patient studies is highly dependent on the location of both the tumor and the node, but generally the lymph node is at the junction of the fatty breast tissue and chest wall.

Superflab (WFR-Aquaplast, Wyckoff, NY) is a tissue-equivalent material used in radiation therapy. Because the material is flexible and its characteristics mimic the scattering properties of tissue, it was used to simulate superficial tissues in the construction of the phantom.

The setup for the thorax phantom study consisted of securing the small spheres directly to the thorax phantom in the anterior axillary region to represent axillary SLNs and near the mediastinum to represent internal mammary SLNs. A 0.5 cm layer of superflab was placed over the small sphere to cushion the objects when the chest overlay was placed onto the thorax phantom. The chest overlay and fillable breast phantoms were placed over the thorax phantom. A cavity just big enough to accommodate the larger sphere (representing the injection site) was formed in a layer of 1.0 cm thick superflab in order to provide a scatter medium for imaging. An additional 1-2 cm of superflab was added as needed to provide scatter similar to that seen in the patient studies of the retrospective chart review for both the anterior and lateral emission images.

Static emission images and CT topograms were acquired for both the anterior and lateral views of the thorax phantom. The images were processed using the geometry compensation program and registered to validate the processing protocol for phantom images that mimic those of patients.

### 2.5. Dosimetry for Each Localization Technique

#### 2.5.1. ^57^Co Backlighting Dosimetry

The photon flux of ^57^Co sheet sources is too low for measurement with routinely used radiographic ion chambers such as a Triad or Radcal because of the insensitivity of the chambers (chamber volumes are not sufficient to detect the low exposure rate of the sheet source). The absorbed dose from a 111 MBq (3 mCi) ^57^Co sheet source was therefore estimated by measuring the exposure rate using a dose-equivalent ion chamber (Inovision 451B-DE-SI, Fluke, Everett, WA). With the gamma camera configured for opposed-head imaging at a gantry angle of zero (i.e., to produce anterior and posterior views of a supine patient), the sheet source was placed on the face of the collimator of the posterior (lower) detector. The ion chamber was placed on the patient couch and centered above the sheet source. Acrylic blocks were placed around the chamber to simulate the scatter in the human torso. The meter readings, in *μ*Sv per hour, represent the dose-equivalent rate at that location. (The 451B-DE-SI conversion factor varies between 0.9 and 1.0 over the range of photon energies present in this setup). The total absorbed dose for unilateral and bilateral breast lymphoscintigraphy studies was calculated by accounting for each view of the study. Unilateral studies had two transmission images acquired whereas bilateral studies had three transmission images acquired.

#### 2.5.2. CT Topogram Dosimetry

The topogram localization images need not be of diagnostic imaging quality. It is most important that the images provide anatomical landmarks by depicting bony anatomy and providing some soft tissue information. Topograms of the anthropomorphic phantom were acquired at 80, 110, and 130 kVp for various mA settings. The images were presented to two physicians who assessed image quality with respect to the ability to adequately localize the hot spheres when fused to the lymphoscintigrams. Anterior and lateral topograms acquired at 110 kVp and 20 mA were deemed to be of sufficient quality for SLN localization. 

The dosimetry for the topogram localization technique was estimated using a 6 cubic centimeter cylindrical ionization chamber (Model 10X5-6) and electrometer (Model 9010) (Radcal Corporation, Monrovia, CA) as described by O'Daniel et al. [18]. The chamber was centered in the gantry of the CT scanner with the stem of the ion chamber (the long axis of the cylinder) parallel to the long axis of the couch. In-air exposure measurements were recorded for both the anterior/posterior (AP) and posterior/anterior (PA) tube positions. The entrance skin exposure was calculated for an average size patient (23 cm anterior-posterior thickness) for each tube position by correcting the isocenter exposure measurement using the inverse-square method. The skin dose was calculated using the Roentgen-to-rad conversion (0.87 rad = 1 R and for SI units 1 rad = 10 mGy) and back-scatter factor (assumed to be 1.35) [19]. In order to properly convert the skin dose measurements to effective dose, a conversion factor of 0.1 mSv per mGy was obtained from the National Radiological Protection Board Report W4 [19].

#### 2.5.3. Computed Tomography (CT) Dosimetry

If CT tomographic images are to be used only to localize SLNs, the CT need not be of diagnostic quality and should be acquired at a relatively low dose (i.e., low mAs). For the purposes of this study, five different scan parameter combinations were tested to assess the range of absorbed doses that might be delivered to the patient: 130 kVp, 90 mAs; 130 kVp, 20 mAs; 110 kVp, 20 mAs; 110 kVp, 10 mAs; and 80 kVp, 20 mAs. 

The CT dose index (CTDI) dosimetry method employs a CTDI body phantom (Model 20CT14) and a 10 cm long, 3 cubic centimeter pencil ionization chamber (Model 10X5-3CT) (Radcal Corporation, Monrovia, CA) [20]. In-phantom measurements of exposure (mR) were recorded for the central chamber position and four peripheral positions. Then, CTDI_100_, CTDI_w_, CTDI_vol_, and dose-length product (DLP) values were calculated according to the definitions in American Association of Physicists in Medicine (AAPM) Report Number 96 [21], using a detector configuration of 6 × 2 mm and pitch of 1.2. The DLP (based on 40 cm scan length) for a given CT scan was converted to effective dose using the conversion factors in the European Guidelines on Quality Criteria for Computed Tomography [22]. Since the localization technique for the breast lymphoscintigraphy protocol has a scan extent throughout the chest region, the *κ*-factor used for this estimation of effective dose was 0.017 mSv per mGy-cm.

## 3. Results

### 3.1. Topogram Spatial Distortion

**Figure 4:**
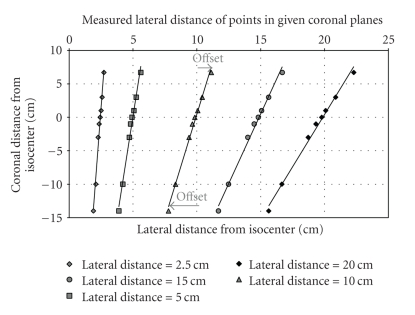
Measured spatial distortion of CT topogram. The error in lateral distance from isocenter (measured-true) of a point object (mammography bead) varied linearly as a function of both the lateral and coronal distance from isocenter.

The measured spatial mismatch between uncompensated scintigrams and topograms increased linearly with distance from the isocenter in the anterior-posterior and lateral directions (Figure 4). The range of measured spatial offset was from 0.0 to 4.4 cm. The measured distance (*x*) of the point sources from the long axis of the topogram when the point source is in a given coronal plane (*β*) can be approximated by (2). The actual lateral distance of the point source from the long axis of the hybrid scanner is denoted by *α*

(2) $$x\left[\text{cm}\right]=\left(0.08\beta +4.98\right)\cdot \left(\frac{\alpha }{5\;\text{cm}}\right).$$

The measured spatial mismatch is calculated by the difference of the measured position of the point source, *x*, and the actual lateral position of the point source from the central axis of the scanner, *α*. The spatial mismatch is estimated by

(3) x[cm]−α[cm]=(0.016β−0.004)·(α).

### 3.2. Verification of the Geometric Compensation

#### 3.2.1. Point Source Phantom

**Figure 5:**
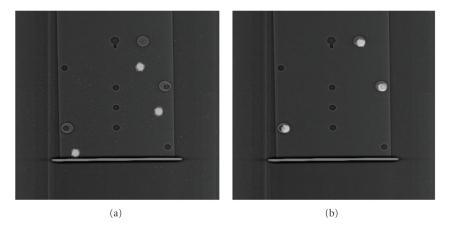
Accuracy of lateral distortion compensation algorithm for planar scintigram-CT topogram registration. Fused images of the original (uncompensated) scintigram and CT topogram of three button sources (a), and stretched (compensated) scintigram and CT topogram (b). The vertical shift in the original fused images is due to a registration offset in the table travel direction of the scanner equal to the distance between the edge of the field of view of the gamma camera detector and the edge of the image matrix. This offset is accounted for in the compensation algorithm so the scintigram and topogram accurately register in the longitudinal direction of the scanner. On the right, the scintigram is compensated to correct the lateral position of the right-most button source. The residual error in lateral position of a given button source after compensation was within ±3.44 mm.

The misregistration between nuclear medicine scintigrams and CT topograms was reduced to within ±3.4 mm in the plane of a particular ^57^Co button source. The residual misregistration is less than the intrinsic resolution of the gamma camera (3.2–3.4 mm) and much less than the overall system resolution of the images. Fused images of the registered, geometrically compensated scintigrams, and topograms of the point source phantom are shown in Figure 5. As the lateral magnification of the topogram is processed separately for each individual object of interest, only the indicated point source is correctly registered since each source is in a different sagittal plane.

### 3.3. Anthropomorphic Thorax Phantom

**Figure 6:**
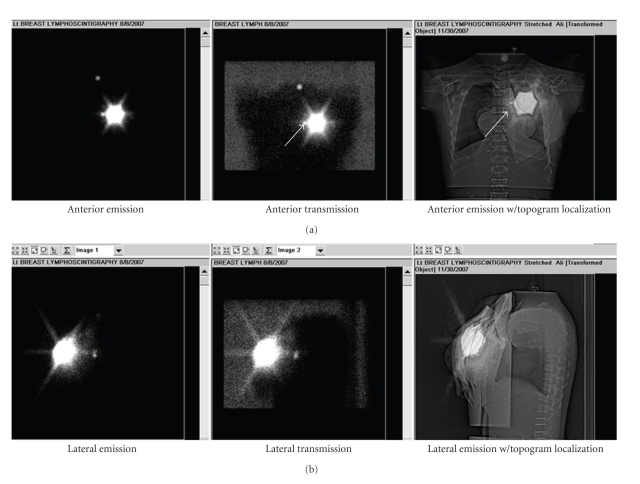
Comparison of scintigram-CT topogram method to conventional lymphoscintigraphy. Anterior (a) and lateral (b) views of ten-minute emission images, conventional localization images, and planar scintigrams fused with CT topograms, of an anthropomorphic thorax phantom with a simulated internal mammary SLN. The current acquisition and processing workflow provides the physician with a visual comparison of the conventional and proposed method of localization. The anterior view of the scintigram-topogram fused image (a-right) provides location information for the internal mammary lymph node relative to the ribs and sternal notches.

In addition to measuring the spatial distortion of topograms by using a point-source phantom, the anthropomorphic thorax phantom was used for qualitative comparison of various lymphoscintigraphic imaging methods. The phantom studies were performed to visually compare our proposed method of localization to the conventional method (Figure 6). The direct comparison of the two techniques illustrates how much additional information is provided by our approach with respect to localizing an SLN relative to bony anatomy.

### 3.4. Dosimetry

#### 3.4.1. ^57^Co Backlighting

 The measured dose rate for a 111 MBq (3 mCi) sheet source was 87 uSv per hour. If the ^57^Co backlit images were acquired for 3 minutes per view, then the estimated effective dose for unilateral and bilateral breast lymphoscintigraphy studies localized with the backlighting technique would be 9 *μ*Sv and 13 *μ*Sv, respectively.

#### 3.4.2. Topogram Localization

The measured in-air exposure at gantry isocenter for a 110 kVp, 20 mA PA topogram was 18 mR, so the exposure at the skin surface would be 19 mR using an inverse-square correction. The estimated skin dose for the CT topogram is 0.23 mGy. Using the 0.1 mSv per mGy conversion from skin dose to effective dose, the estimated effective dose for the 110 kVp, 20 mA topogram would be 22.5 *μ*Sv. NRPB-W4 does not provide an mSv per mGy conversion from skin dose to effective dose for lateral exams. However, based on several other known acquisition techniques and configurations, it is reasonable to expect that the effective dose for the lateral topogram would be only one order of magnitude greater than the estimated dose using ^57^Co backlighting (Table 1).

#### 3.4.3. CT Localization

The effective dose to the chest for a CT scan ranged from 0.308 mSv for the lowest possible dose setting (80 kVp, 20 mAs) to 5.265 mSv for the default clinical CT scan setting (130 kVp, 90 mAs). The calculations for each dose index, the DLP, and the effective dose for each of the CT scan parameter combinations are compiled in Table 2.

## 4. Discussion

The fusion of a geometry-compensated lymphoscintigram with a CT topogram is a new method of anatomical localization for lymphoscintigraphy. When compared to the time-honored means of localization using backlighting with a ^57^Co sheet source, it offers a substantial increase in both the number and detail of anatomical references at the relative detriment of an order of magnitude increase in the still quite modest absorbed dose to the patient. Furthermore, the gantry of the SPECT/CT camera can be rotated to acquire the lateral topogram and the lateral scintigrams while the patient does not move. Thus all of the views may be acquired without moving the patient from a position close to that in which subsequent biopsy or surgery would be performed. While a full CT exam would avoid the need for geometrical compensation, it would impose a further increase of one to two orders of magnitude over our proposed approach in absorbed dose to the patient.

The software program used to adjust the scintigrams was written in IDL in such a fashion that it functions stand-alone or it can be incorporated into a vendor image processing workstation using workflow “broker activities.” The image registration and fusion of the stretched scintigrams with “NM” DICOM tags and CT topograms with “NM” DICOM tags can be performed with any vendor software program that registers and fuses two planar nuclear medicine (NM) images. Although the IDL program was integrated into the Siemens E.Soft image processing workstation for this project, the images were also fused successfully using OsiriX (Pixmeo, Switzerland). 

The most fruitful cases in which to conduct the first clinical evaluations of this technique would be in regions of the body of moderate complexity such as for drainage to the internal mammary lymphatic chain, where the surgeon must know which rib to remove in order to access a given node. Extremely complex anatomy, such as the head and neck, calls for a full SPECT/CT study, while in cases of drainage to the axillary lymphatic chain, the plasticity of the tissues and the absence of skeletal interference to the surgical approach make the improvement of topogram fusion over sheet source backlighting of less value.

## 5. Conclusion

Gamma camera images of the phantom described herein are realistic-looking surrogates for actual patient lymphoscintigrams. The phantom meets the need for a reproducible object in which the true location and activity of a simulated lymph node is known.

We demonstrated that fusing lymphoscintigrams to CT topograms is superior to ^57^Co backlighting for producing detailed anatomic localization images. Through our approach, simulated lymph nodes in anterior and lateral scintigrams can be localized to within ±3.4 mm between the fused scintigram and topogram. This misregistration error is less than the intrinsic resolution, and much less than the system compound resolution, of the gamma camera for lymphoscintigraphic imaging. 

Moreover, for many clinical applications, fusion of a planar scintigram with a CT topogram may offer the best compromise between absorbed dose to the patient and critical anatomic detail.

## Figures and Tables

**Table 1:** Measured exposure and calculated CT topogram dose versus kV and mA. For topogram acquisitions, three conversion factors (Rontgen-to-mGy, backscatter factor, and skin dose-to-effective dose) are used to estimate the effective dose for several techniques of topogram acquisition.

KV	mA	Time [s]	Tube position	Measured exposure (in-air) [mR]	Skin dose [mGy]	Effective dose (NRPB-W4) [mSv]
130	20	3.1	Top (A/P)	32.29	0.40	0.040
			bottom (p/A)	25.91	0.32	0.032
110	20	3.1	top (A/p)	23.19	0.28	0.028
			Bottom (P/A)	18.36	0.23	0.023
80	20	3.1	Top (A/P)	12.72	0.16	0.016
			Bottom (P/A)	9.78	0.12	0.012

**Table 2:** Measured exposure and calculated CT dose versus kVp and mAs. In CT scans, the CTDI_vol_ and DLP values are used to convert in-phantom ion chamber measurements to estimated effective dose in the standard CT dosimetry phantoms (*μ*Sv).

KVp	mAs	location	Exposure [R]	CTDI__vol_ [mGy]	DLP [mGy∗cm]	Effective dose [mSv]
80	20	center	0.00395	0.453	18.137	0.308
		Periphery	0.00928			
110	10	center	0.00588	0.576	23.057	0.392
		Periphery	0.01137			
110	20	center	0.01177	1.153	46.115	0.784
		Periphery	0.02274			
130	20	center	0.01791	1.720	68.818	1.170
		Periphery	0.03376			
130	90	center	0.08059	7.742	309.681	5.265
		Periphery	0.15192			
